# Design Space Identification and Visualization for Continuous Pharmaceutical Manufacturing

**DOI:** 10.3390/pharmaceutics12030235

**Published:** 2020-03-05

**Authors:** Samir Diab, Dimitrios I. Gerogiorgis

**Affiliations:** School of Engineering, Institute for Materials and Processes (IMP), University of Edinburgh, The King’s Buildings, Edinburgh EH9 3FB, Scotland, UK; S.Diab@ed.ac.uk

**Keywords:** Continuous Pharmaceutical Manufacturing (CPM), Active Pharmaceutical Ingredients (APIs), design space investigation, technoeconomic analysis

## Abstract

Progress in continuous flow chemistry over the past two decades has facilitated significant developments in the flow synthesis of a wide variety of Active Pharmaceutical Ingredients (APIs), the foundation of Continuous Pharmaceutical Manufacturing (CPM), which has gained interest for its potential to reduce material usage, energy and costs and the ability to access novel processing windows that would be otherwise hazardous if operated via traditional batch techniques. Design space investigation of manufacturing processes is a useful task in elucidating attainable regions of process performance and product quality attributes that can allow insight into process design and optimization prior to costly experimental campaigns and pilot plant studies. This study discusses recent demonstrations from the literature on design space investigation and visualization for continuous API production and highlights attainable regions of recoveries, material efficiencies, flowsheet complexity and cost components for upstream (reaction + separation) via modeling, simulation and nonlinear optimization, providing insight into optimal CPM operation.

## 1. Introduction

Increasing pharmaceutical Research and Development (R&D) expenditures necessitate the need for leaner manufacturing with reduced costs. There has been significant research focus on continuous Active Pharmaceutical Ingredient (API) production due to pressure on the pharmaceutical industry to reduce drug development times, minimize product quality variation and process deviations, decrease overall costs and minimize environmental impact with lower capital and operating expenditures that are inherent of the smaller equipment and material usage reductions with continuous operations [1,2]. The chemistry, chemical engineering and process systems engineering communities have approached both unit operation and plantwide Continuous Pharmaceutical Manufacturing (CPM) from both experimental (laboratory and pilot plants) and theoretical (mathematical modeling, simulation and optimization) perspectives to elucidate promising designs for continuous API production [3,4].

Design space investigation for different process options is a useful task in elucidating critical process parameters as well as understanding the attainable region of product quality attributes and technoeconomic performances. Our group has conducted many studies in technoeconomic modeling, simulation and optimization of CPM for a variety of APIs, including both upstream plants (flow synthesis + purification/separation) and isolated separation trains (e.g., crystallization cascades). An understanding of the attainable performances within design spaces for different APIs that have been realized as amenable to CPM (from studies in the literature) can be useful in elucidating technoeconomic viability vs. existing processes. Therein lies the novelty of this study.

This paper is structured as follows. First, we discuss the need for design space investigation and visualization for CPM design, followed by a review of some pertinent literature, including API flow synthesis, purification/separation and downstream unit operations. We then consider upstream CPM case studies conducted by our group, which have utilized conceptual modeling, simulation and optimization—Nonlinear Programming (NLP) and Mixed Integer Nonlinear Programming (MINLP)—for comparative technoeconomic evaluation of CPM designs. A critical discussion of these cases is then provided with an outlook to the future of this vibrant research field.

## 2. Related Literature

This section is structured as follows. First, we discuss different stages of pharmaceutical manufacturing and literature studies (experimental, industrial and theoretical) on continuous applications: Section 2.1 discusses API continuous flow synthesis, Section 2.2 considers continuous flow synthesis and purification + separation process design and Section 2.3 focuses on examples of downstream processing and Drug Product (DP) formulation. Subsequently, Section 2.4 discusses the industrial adoption of CPM and the need for an understanding of regions of attainable performances of APIs that have been demonstrated as amenable to CPM. Section 2.5 then discusses design space investigation for CPM, how modeling, simulation and optimization methodologies can be used to rapidly screen process designs and elucidate attainable regions, and describes literature examples of design space investigation for different stages of CPM. Section 2.6 then summarizes the relevant literature discussed and provides the motivation, aims and objectives of this paper.

### 2.1. Continuous Flow Synthesis of Active Pharmaceutical Ingredients

The development of continuous flow technology and synthetic strategies by chemists and engineers has been the focus of significant research attention over the past two decades due to the wide variety of chemical processes whose performance can be improved or intensified by switching from batch to continuous flow operation. While continuous manufacturing is the norm in many process industries (e.g., oil and gas production), continuous operation is less frequented in pharmaceutical manufacturing due to significant investments in batch infrastructures and various other advantages [5]. Operating continuously allows for smaller equipment dimensions, wherein mixing and heat transfer are significantly enhanced, improving yields, selectivities, productivities and allowing access to novel operating windows that would be otherwise hazardous in batch mode [6]. Several reviews in the past few years have documented the rapid development of continuous flow synthesis technology for fine chemical and pharmaceutical products [7,8,9,10,11,12].

### 2.2. Continuous Pharmaceutical Separation Process Design

The development of continuous separation processes as part of end-to-end CPM plants is essential for reaction effluent purification and crystallization prior to downstream unit operations. While some demonstrations of fully continuous plants (including synthesis, purification and drug product formulation) have been highlighted in the past decade [13,14,15], there is generally a lack of continuous separation process design in the literature in comparison to research efforts in flow synthesis [16], especially as part of integrated plants. That said, there have been investigations into the design, operation and optimization of isolated continuous pharmaceutical separation processes.

A variety of different separation processes are implemented in pharmaceutical manufacturing processes, including (but not limited to) Liquid–Liquid Extraction (LLE), membrane purification/separation, distillation, crystallization and chromatographic separations. Modeling and optimization have been implemented in the design of continuous chromatographic methods [17] and membrane separations [18] for pharmaceutical manufacturing. Here, we focus on the design of continuous LLE and crystallization processes, both of which are very commonly implemented purification and separation processes essential to pharmaceutical manufacturing.

#### 2.2.1. Liquid-Liquid Extraction

The aim of LLE is to purify a multicomponent mixture by addition of a solvent which induces the splitting of the mixture into multiple phases, between which solutes partition; the objective is to partition undesired solute component (e.g., impurities) into one phase while the desired solute (e.g., product API) preferentially partitions into the other. Purification via LLE is typically implemented in pharmaceutical processes prior to crystallization to ensure as few undesirable impurities as possible are incorporated into crystalline products. The design of continuous LLE processes is an important aspect of end-to-end CPM plant development.

The majority of LLE process demonstrations are still done in batch, even with a continuous flow synthesis demonstration. That said, there have been a few experimental as well as theoretical studies on the design of continuous LLE processes. Drageset and Bjørsvik (2016) performed an in-line continuous LLE for purification of a reactor product mixture prior to further downstream processing, allowing for significant material reduction compared to the batch purification process [19]. Monbaliu et al. (2016) also implemented a continuous LLE process as part of an end-to-end CPM process for lidocaine hydrochloride (a local anesthetic) from synthesis to aqueous formulation [20]. Implementation of combined experimental and modeling approaches towards integrated LLE design described in the literature for pharmaceutical purifications and separations demonstrate the utility of theoretical methods in establishing optimal design and operating parameters [19,20,21].

#### 2.2.2. Crystallization

A significant portion of pharmaceutical products are sold as solids (tablets, dispersions, gels or topical treatments), and thus crystallization is an essential unit operation in Drug Product (DP) manufacturing. The aim of crystallization is to form a solid product of the desired compound with minimal impurity + solvent incorporation into the crystal structure while also attaining the desired polymorph, suitable mean crystal size and size distribution properties, all of which affect subsequent downstream process unit operations and the bioavailability of the drug in the patient.

Continuous crystallization has received attention for its potential to increase flexibility, efficiency and quality [22]. Continuous crystallization operates under steady-state conditions, allowing higher reproducibility and better control of important crystal properties such as the purity and the size distribution; however, as continuous processes do not discharge at equilibrium, they tend to achieve lower yields than batch crystallizations [23]. Continuous crystallizer designs applicable for the pharmaceutical industry are categorized as Plug Flow Crystallizers (PFCs), Continuous Oscillatory Baffled Crystallizers (COBCs) or Mixed Suspension–Mixed Product Removal (MSMPR) crystallizers. PFCs are suited to systems with fast crystal growth and short residence times and can attain narrow crystal size distributions [24], but fouling and clogging in narrow tube diameters is an important technical issue [25]. COBCs are another emerging technology, which enhance heat and mass transfer, but have issues handling streams with high solid loadings [26]. Various experimental and modeling studies have been conducted for the estimation of crystallization kinetics, proof-of-concept demonstrations and design and optimization processes [27,28,29,30,31,32].

### 2.3. Continuous Downstream Processing + Drug Product Formulation

Pharmaceutical manufacturing can be classified into distinct stages in series: synthesis, the single- or multistep reaction to form the target API molecule from reagents; purification and separation, the removal of API/impurities from the reaction mixture to sufficient purity that is often followed by crystallization due to most drugs being administered as solids; and formulation, where the Drug Product (DP) is made, making the API into a form that is consumable and effective.

Downstream processing for DP formulation is an essential set of stages in a pharmaceutical plant in order to yield the final product in the form by which it will be administered. As such, stringent product quality assurance in these stages is paramount. Continuous manufacturing in these stages is required for successful end-to-end CPM implementation (i.e., all stages of the plant are continuous without intermediate batch processing required).

Various unit operations exist for the formulation of different types of DPs, depending on the form in which it is to be administered to the patient. Different studies consider varying flowsheet complexities and combinations of unit operations, a few pertinent literature examples of which are briefly described here. Su et al. (2019) implemented model predictive control to attain specific product quality attributes that were met for a rotary tablet press (composed of filling, metering, compression and tablet ejection) as part of a downstream pilot plant [33]. Martinetz et al. (2017) also considered a similar continuous rotary tablet press, which was designed to be robust with respect to varying feed flow rates for different product formulations [34]. Metta et al. (2019) performed design and dynamic simulation of a continuous tableting line (composed of feeders, blenders, wet granulation, fluidized bed drying, milling + tablet press). The authors elucidated the critical process parameters affecting product quality that needed to be ensured in order to facilitate robust process design [35].

Hsu et al. (2010) formulated a dynamic model for continuous roller compaction using a model based approach which was subsequently used for control due to the process being very sensitive to inlet bulk density [36,37]. Bano et al. (2019) also considered the continuous roller compaction of microcrystalline cellulose, combining first-principles methods with plant data to establish the alteration of the design space subject to process disturbances and uncertainty [38]. Tian et al. (2019) considered a continuous direct compression line (feeders, blenders + press) which was modelled and simulated following a sensitivity analysis for the elucidation of critical process parameters [39].

### 2.4. Industrial Adoption of Continuous Pharmaceutical Manufacturing

While manufacturing sectors such as the oil and gas industry traditionally operate in continuous mode, pharmaceutical production is traditionally batchwise, only implementing continuous mode for select cases in the past decade. Continuous manufacturing was highlighted as a key green chemistry principle by various industrial and regulatory bodies [1,40], with various efforts towards generalizing methodologies towards facilitation of the transition of batch to continuous processes [2,3]. That said, the selection of which processes can be conducted continuously is still predominantly done on a case-by-case basis. Here, we list some examples of adoption of continuous manufacturing for different pharmaceutical processes at different scales.

The first fully end-to-end CPM plant was demonstrated by Mascia et al. (2013), where aliskiren hemifumarate was synthesized, purified, crystallized and subsequently formulated into the desired DP whilst mitigating solids handling issues and removing the need for intermediate solvent swaps between unit operations [41]. Adamo et al. (2016) also demonstrated a compact, end-to-end production of multiple DPs with different APIs [13]. Monbaliu et al. (2016) developed an automated system for the end-to-end CPM of lidocaine hydrochloride [20]; Cole et al. (2019) described the end-to-end CPM of merestinib (a new biliary tract cancer drug) from synthesis to crystallization [14,15]. The above-mentioned demonstrations were implemented on pilot or production plant level.

The implementation of CPM at production level has begun to appear more prevalently in recent years for plant subsystem and plantwide designs [42,43,44]. Vertex committed to continuous Orkambi (containing lumacaftor and ivacaftor for cystic fibrosis treatment) tableting [45]. GSK began continuous production of amoxicillin at a fully continuous plant in Singapore [46] and also began a continuous line for daprodusat (a new anemia mediation) [47,48]. In 2016, Janssen received FDA approval for the continuous tablet production for Prezista, whose API, darunavir, is used as part of HIV/AIDS combinative treatments [49]. Eli Lilly recently committed a significant capital investment to a continuous production plant in Ireland [50].

The relatively few examples of industrial adoption of CPM highlights a need for understanding the attainable process performances of candidate designs to ensure CPM success; this is where design space investigation via modeling, simulation and optimization can be useful tools.

### 2.5. Design Space Investigation

The literature contains many examples of design space investigation studies for both batch and CPM processes on the unit operation, subsystem or plant level. Here, we describe some pertinent examples of such studies in flow synthesis, separation and downstream processing.

#### 2.5.1. Continuous Flow Synthesis

The demonstration of continuous flow chemistry of an API (i.e., in which the synthesis is performed in flow as opposed to in batch) is the foundation of any CPM process; however, subsequent purification + separation (upstream) and DP formulation (downstream) unit operations are often challenging and expensive processes that must be considered in the comparative evaluation of different designs. Establishing feasible operating regions to meet desired product quality and process performance targets is an important part of design in various CPM studies.

Process chemists often spend extensive periods of time attempting to find optimal reaction conditions (temperature, residence time, reagent and/or base concentration, catalyst loading etc.) in order to maximize product yields, selectivities and purities. Whilst a mechanistic understanding of chemical reactions is useful (via development of deterministic models that can be reparameterized for use in other similar reaction systems), their development can be time-consuming and a data-driven approach may be more convenient for the specific application. However, retrieving sufficient data to have a meaningful data-driven model can be laborious and time-consuming in itself. For this reason, the development of automated continuous flow systems for reaction is a hot topic of research.

**Figure 1:**
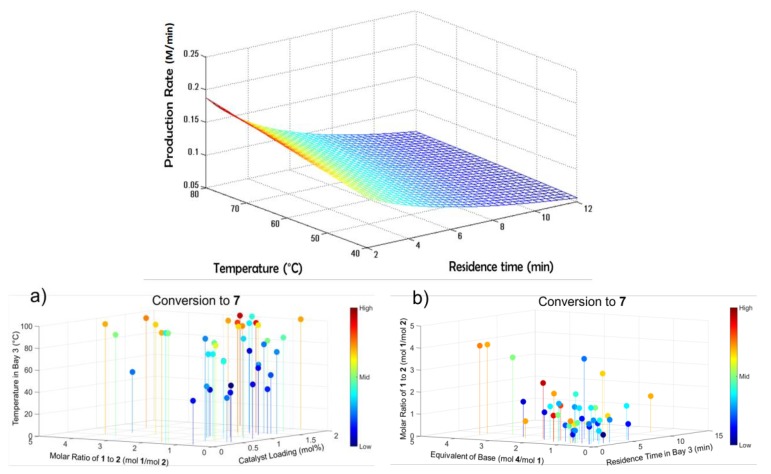
Reaction design space mapping in a reconfigurable continuous synthesis system: (**a**) Attained conversion vs. reagent molar ratios, catalyst loading and operating temperature, (**b**) Attained conversion vs. base equivalents, residence time and reagent molar ratios. Reproduced with permission from Bédard et al., Science; published by American Association for the Advancement of Science, 2018 [51].

Bédard et al. (2018) developed a continuous synthesis system composed of reagent/feedstocks and pumps and interchangeable reactor and separator modules with online analytics and a software interface for process control and reaction monitoring (Figure 1). The authors demonstrated a variety of pharmaceutical reactions in flow, elucidating optimal regions of operation regarding operating temperature, residence time, reagent ratios, catalyst and base loading [51]. The modularity and automated nature of the system allowed for optimal reaction conditions being elucidated for C–C and C–N cross-couplings, olefinations, reductive aminations nucleophilic aromatic substitution, photoredox catalysis and multistep sequences thereof, all of which are highly relevant to the flow chemistry community, particularly for the continuous synthesis of APIs.

**Figure 2:**
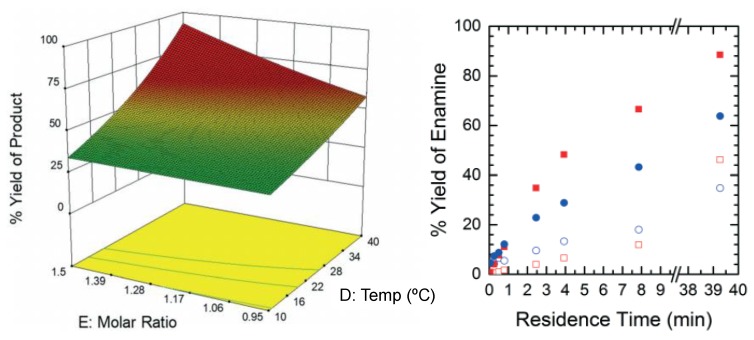
Design space investigation of the flow synthesis of an intermediate towards dolutegravir. Reproduced with permission from Armstrong et al., Reaction Chemistry & Engineering; published by Royal Society of Chemistry, 2019 [52].

Armstrong et al. (2019) performed design space investigation to understand the effect of the critical operating parameters (reagent molar ratios and reaction temperature) on the flow synthesis of an intermediate to dolutegravir, a HIV integrase inhibitor used in combinative HIV/AIDS treatment (Figure 2) [52]. The authors also compared the performance and accuracy obtained by design space investigation using a Computational Fluid Dynamics (CFD) approach vs. experiments, with the CFD results being comparably accurate but faster and without experimental labor.

Reizman and Jensen (2015) considered the effect of reaction carrier solvent for optimization of a synthesis performed in microliter slugs, comparing the process yield as a function of reagent molar ratios and residence time in each considered solvent [53]. The authors screened discrete (reactor materials, catalyst, base, carrier solvent) and continuous (reaction temperature, residence time) variables simultaneously; their automated system was used to maximize reaction yield. The study is in a similar vein to Bédard et al. (see Figure 1), but without the automated experimental setup, which is not a commercially available rig.

**Figure 3:**
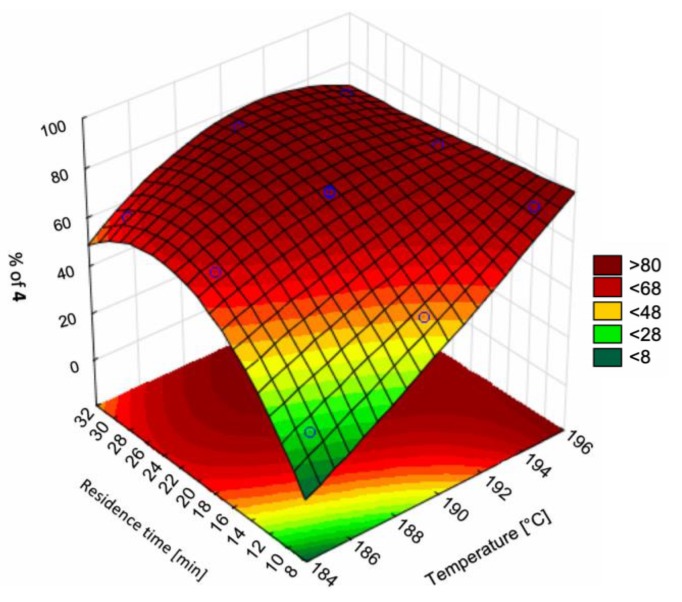
Reaction optimization for vortioxetine synthesis. Reproduced with permission from Boros et al., Journal of Flow Chemistry; published by Springer Nature, 2019 [54].

Boros et al. (2019) considered the flow synthesis of vortioxetine (an antidepressant API) from an intermediate, comparing the effects of temperature and residence time on process yield [54]. Their design space investigation vs. different batch configurations showed improvements with respect to product yield and purity, illustrating the benefit of design space investigation in order to maximize the benefits of CPM compared to existing batch processes (Figure 3).

**Figure 4:**
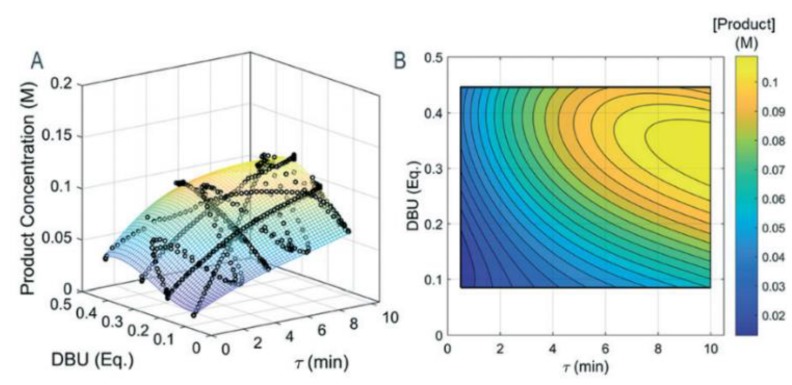
Flow synthesis design spaces of a Knoevenagel condensation in flow as (**A**) 3D projection with experimental data points, (**B**) 2D projection. Reproduced with permission from Wyvratt et al., Reaction Chemistry & Engineering; published by Royal Society of Chemistry, 2019 [55].

Wyvratt et al. (2019) characterized the design space of a Knoevenagel condensation (a nucleophilic addition of a carbanion to a carbonyl compound followed by dehydration, which is a key reaction stage in the production of many APIs). The authors elucidated the design space by varying residence time and catalyst loading whilst minimizing the number of experiments and material consumption required to adequately map the design space (Figure 4). The number of experiments, and hence material requirements, needed for data-driven reaction modeling is one of the main drawbacks of the approach; hence, the study provides a valuable methodology for materially-efficient design space elucidation for flow synthesis [55].

**Figure 5:**
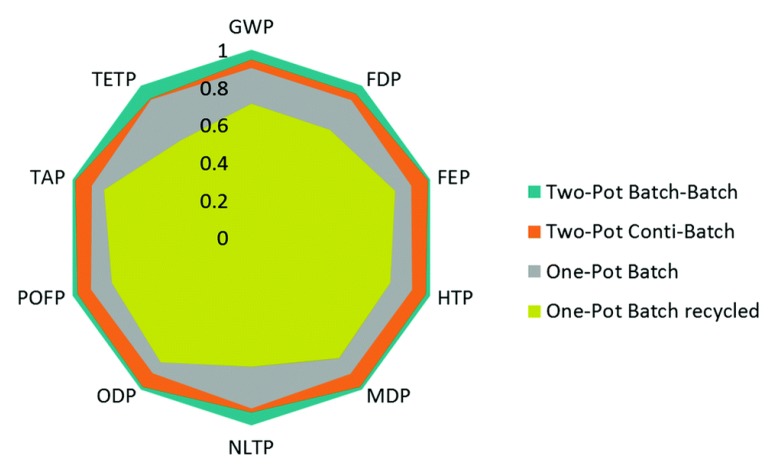
Life Cycle Assessment (LCA) of batch vs. continuous synthesis for rufinamide. Reproduced with permission from Ott et al., Green Chemistry; published by Royal Society of Chemistry, 2016 [56].

Comparative evaluation of batch vs. continuous syntheses are also useful in quantifying technoeconomic benefits of different production paradigms and flowsheet configurations [55]. Life Cycle Assessment (LCA) has been performed for different flow chemistry and plant design studies in the literature for pharmaceuticals and the production of other chemicals. Ott et al. (2016) performed LCA for different flowsheet configurations of batch vs. flow microreactor networks for rufinamide synthesis, considering various metrics related to plant material efficiencies and environmental impacts (including potentials for global warming, human toxicity, natural land transformation, ozone depletion, photochemical oxidation and terrestrial acidification and ecotoxicity) of different production options (Figure 5) [56]. The authors found that each of the considered batch/continuous processes presented inherent trade-offs between different LCA criteria and process chemistry options. This study (amongst others) demonstrates typical trade-offs and complexity in process synthesis and design selection, which can be aided by detailed design space investigations.

#### 2.5.2. Continuous Separation Process Design

**Figure 6:**
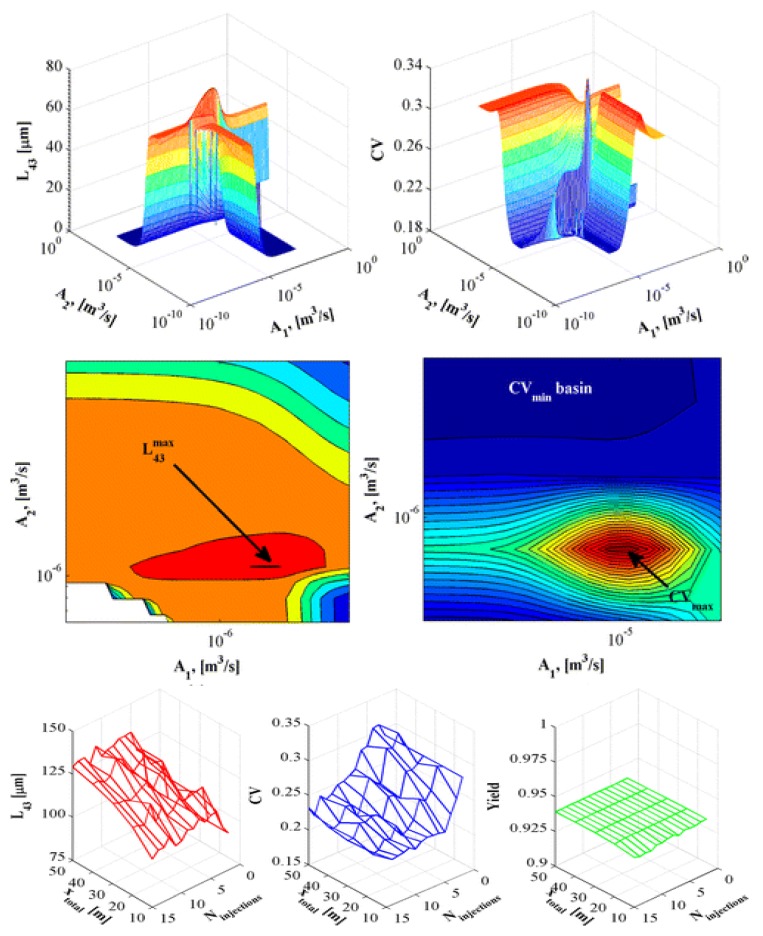
Design space of crystal quality (average size + size distribution coefficient of variation) vs. antisolvent addition at different inlets. Reproduced with permission from Ridder et al., Industrial & Engineering Chemistry Research; published by American Chemical Society, 2014 [57].

In crystallization processes, critical product quality attributes (mean product size and size distribution width) that affect downstream processing and drug bioavailability are very sensitive to process design and operating parameters. Ridder et al. (2014) performed experiments and modelled the antisolvent crystallization of flufenamic acid in a multisegment, multiaddition plug-flow crystallizer, where the antisolvent feed rate to different tubular crystallizer segments was varied in order to either maximize the mean crystal size or minimize the product size distribution coefficient of variation (Figure 6). The authors presented trade-offs between the two product quality attributes [57]. The study demonstrated the benefits of rigorous modeling and optimization for process design whilst circumventing expensive experiments to attain specific quality attributes.

**Figure 7:**
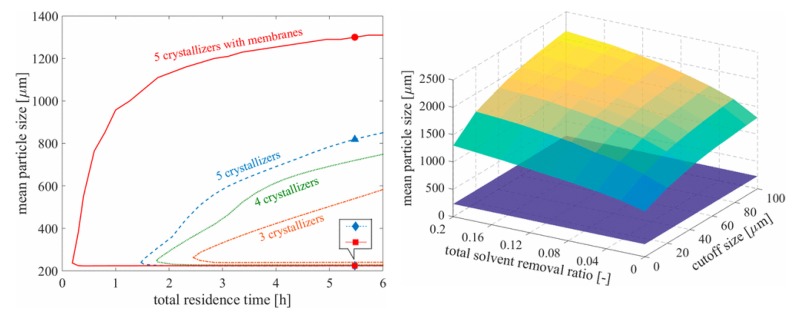
Attainable crystal particle size as a function of number of vessels, residence time, solvent removal ratio and cutoff size. Reproduced with permission from Wang and Lakerveld, Industrial & Engineering Chemistry Research; published by American Chemical Society, 2017 [18].

In the past decade, process integration and intensification have been recent topics of interest within the process chemistry and systems engineering communities for their ability to attain otherwise difficult windows of product quality whilst also minimizing equipment dimensions and material consumption. Wang and Lakerveld (2017) combined a membrane separation with a MSMPR crystallization cascade with mother liquor recycle to maximize particle size by varying crystallizer operating temperatures subject to impurity limits, temperature constraints, yield specification, set cascade residence time and solvent removal rate [18]. Their results showed that incorporating membrane separations into a traditional MSMPR cascade significantly widens the region of attainable product particle sizes whilst shortening the total residence time (Figure 7). The regions of attainable product quality were shown to be significantly larger when implementing longer crystallization cascades with membranes vs. those without membranes.

**Figure 8:**
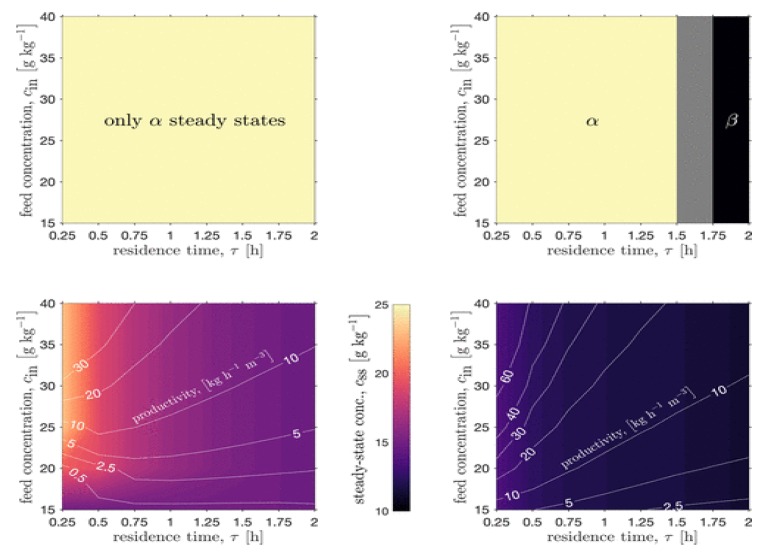
Process intensification of continuous crystallization + milling for polymorph selection. Reproduced with permission from Köllges and Vetter, Organic Process Research & Development; published by American Chemical Society, 2019 [58].

Köllges and Vetter (2019) designed a single MSMPR crystallizer coupled with milling to attain the stable β-polymorph of l-glutamic acid from aqueous solution, mapping the attainable process productivities vs. polymorphic regions (Figure 8) [58]. The authors showed that only via addition of a milling process to the crystallization process allowed for attainment of the β-polymorph, whereas the crystallizer alone could only produce the metastable α-polymorph. The design space study elucidated the fact that the fines generation from milling increased the available crystallization surface area, which enhanced the yield and widened the attainable region of product quality attributes vs. the crystallization process alone.

#### 2.5.3. Downstream Processing

**Figure 9:**
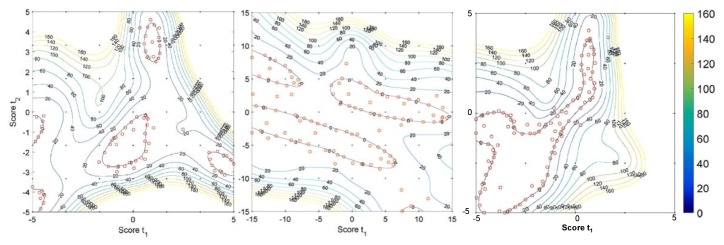
Design space characterization of a milling + tableting process. Reproduced with permission from Bano et al., Computers and Chemical Engineering; published by Elsevier, 2018 [60].

Various efforts towards design space investigation and characterization of downstream pharmaceutical unit operations exist in the literature. Many of these studies make use of statistical techniques such as Partial Least Squares (PLS) and Principal Component Analysis (PCA) methods to understand the underlying causalities and correlations between various plant input and output variables and process parameters, especially when there are significant historical data available and insufficient time to develop, parameterize and validate more detailed mechanistic models [59]. Bano et al. (2018) elucidated the design spaces for three different pharmaceutical process case studies of different size (including a blending and tableting subprocess) and complexity using PLS and applying a Radial Basis Function (RBF) to define the process’ feasible region (Figure 9) [60].

**Figure 10:**
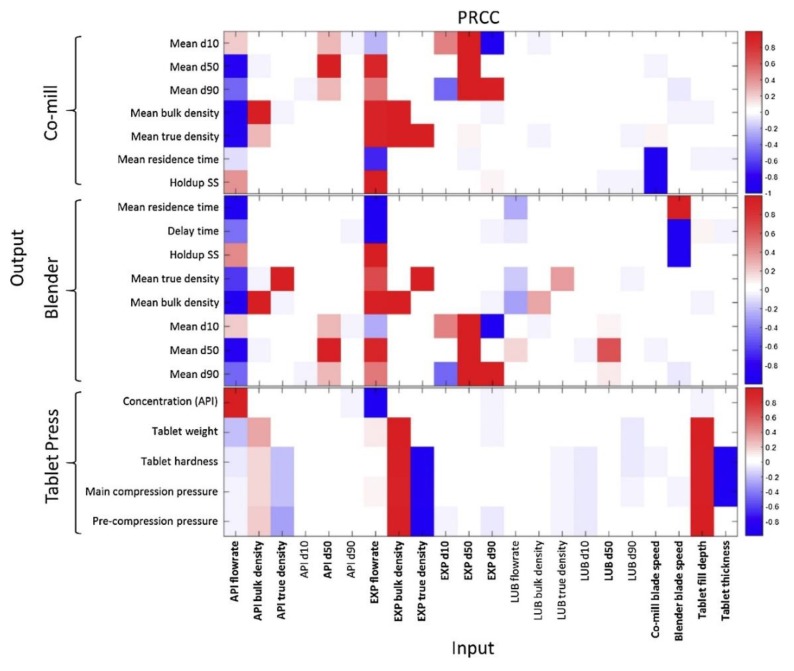
Process sensitivity analysis to aid design space investigation. Reproduced with permission from Wang et al., Computers and Chemical Engineering; published by Elsevier, 2017 [61].

Wang et al. (2017) considered the design space of a milling + blending + tableting process with many dimensions (problem variables), including stream flowrates, target densities, unit residence times, holdups, product particle size distribution properties and final table properties such as weight, hardness and API concentration [61]. Performing a sensitivity analysis (via Morris Screening) on the effects of inputs vs. outputs aided the elucidation of the feasible region of operation (Figure 10).

### 2.6. This Study

It has been illustrated in the above subsections discussing the literature and the state of continuous manufacturing in the pharmaceutical industry that design space investigation via modeling, simulation and optimization can be of great utility for CPM development. In this work, we focus on the upstream CPM of several APIs for which technoeconomic simulation and optimization studies have been performed in the literature for comparative evaluation of typical attainable process performances. Observation of trends common between different APIs, despite their widely varying processes and chemistries, provides a deeper understanding of the attainable process performances for APIs which are amenable to CPM and can be done so successfully.

## 3. Plantwide Design Space Investigation

In this study, we concentrate on upstream plantwide CPM studies we have previously done, encompassing both reaction (flow synthesis) and separation (continuous LLE or crystallization) phenomena and unit operations as well as detailed Capital (CapEx) and Operating (OpEx) Expenditure cost components.

### 3.1. Upstream Plantwide Design Case Studies

The following APIs are considered for analysis in this study: ibuprofen (the popular analgesic), artemisinin (a potent antimalarial), diphenhydramine (a branded antihistamine), warfarin (an anticoagulant), atropine (treatment of nerve agent effects) and nevirapine (used in HIV treatments).

#### 3.1.1. Ibuprofen

**Figure 11:**
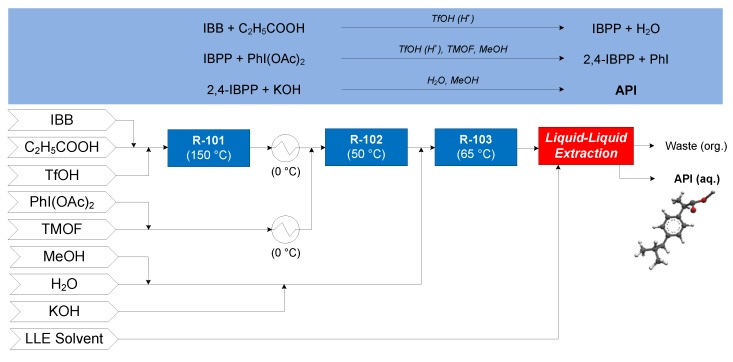
Continuous Pharmaceutical Manufacturing (CPM) flowsheet for ibuprofen: flow synthesis [62] + continuous Liquid–Liquid Extraction LLE [63]. Reproduced with permission from Jolliffe and Gerogiorgis, Computers and Chemical Engineering; published by Elsevier, 2016.

The continuous flow synthesis of ibuprofen was demonstrated by Bogdan and coworkers (2009), consisting of three consecutive reactions in flow [62], followed by a conceptual continuous LLE process [63]—the CPM flowsheet for this process is shown in Figure 11. Isobutylbenzene (IBB), propanoic acid and neat triflic acid (TfOH, catalyst) enter reactor R-101 where IBB undergoes Friedel–Crafts acylations to form 1-(4-isobutylphenyl)propan-1-one (1-4-IBPP). After cooling the effluent of R-101 to 0 °C, it is mixed with a stream of diacetoxyiodobenzene (PhI(OAc)_2_) and trimethyl orthoformate (TMOF) in methanol (MeOH, at 0 °C) prior to entering R-102, where IBPP undergoes 1,2-aryl migration to form 2-(4-isobutylphenyl)propanoate (2-4-IBPP) by catalysis from TfOH. Potassium hydroxide (KOH) in MeOH + H_2_O is added to the effluent of R-102; the resulting mixture enters R-103, where 2-4-IBPP is saponified to form the potassium salt of ibuprofen. The continuous LLE of ibuprofen from the mixture compares toluene (PhMe) and *n*-hexane (nHex) as LLE solvents.

#### 3.1.2. Artemisinin

**Figure 12:**
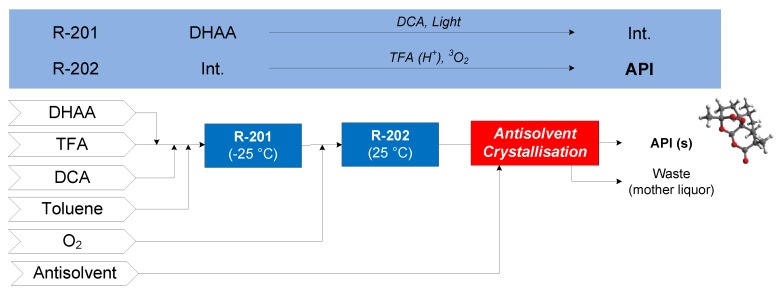
CPM flowsheet for artemisinin: flow synthesis [64] + continuous LLE [63]. Reproduced with permission from Jolliffe and Gerogiorgis, Computers and Chemical Engineering; published by Elsevier, 2016.

The continuous flow synthesis of artemisinin considered is that demonstrated by Kopetzki et al. (2013), where dihydroartemesinic acid (DHAA) is photoxidized to an intermediate (Int.) by the photocatalyst dicyanoanthracene (DCA) in R-201 [64]. Various reactions then occur in R-202—the desired pathway is where the intermediate from R-201 is acid catalyzed (by trifluoroacetic acid, TFA) to produce another intermediate via terminal protonation, followed by a Hock arrangement into another intermediate, which can then react with triplet oxygen (^3^O_2_) to form artemisinin. The effluent of R-202 is neutralized, followed by purification and antisolvent addition followed by cooling to crystallize artemisinin [63]. Ethanol (EtOH) and ethyl acetate (EtOAc) are compared as candidate crystallization antisolvents. The conceptual flowsheet for artemisinin CPM is shown in Figure 12.

#### 3.1.3. Diphenhydramine

**Figure 13:**
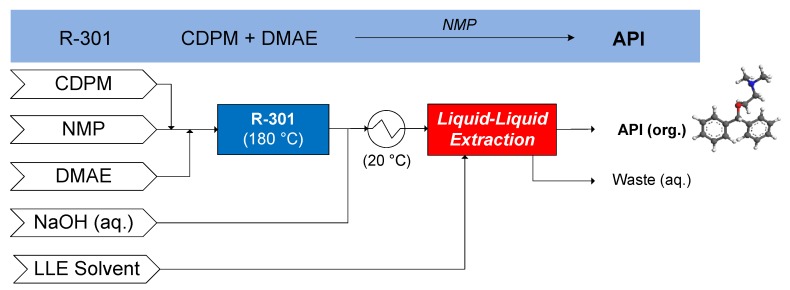
CPM flowsheet for diphenhydramine: flow synthesis [65] + continuous LLE [66]. Reproduced with permission from Diab and Gerogiorgis, Organic Process Research & Development; published by American Chemical Society, 2017.

The continuous flow synthesis of diphenhydramine was demonstrated by Snead and Jamison (2013), wherein chlorodiphenylmethane (CDPM) reacts with dimethylaminoethanol (DMAE) in *N*-methlypyrrolidone (NMP) carrier solvent at 180 °C (R-301) [65]. Subsequently, continuous LLE is performed, comparing cyclohexane (CyHex), methylcyclohexane (MeCyHex) and *n*-heptane (nHep) as candidate LLE solvents, performing the LLE at 20 °C [66]. The conceptual CPM flowsheet for diphenhydramine is shown in Figure 13.

#### 3.1.4. Warfarin

**Figure 14:**
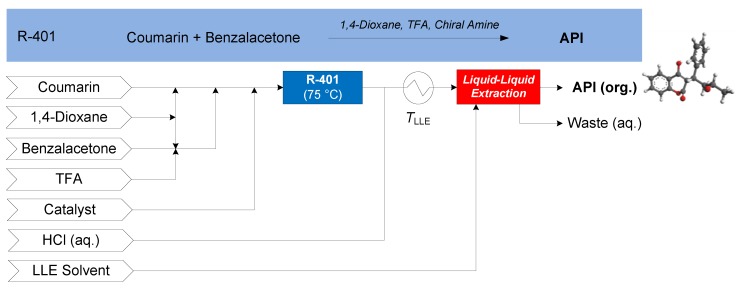
CPM flowsheet for warfarin: flow synthesis [67] + continuous LLE [68]. Reproduced with permission from Diab and Gerogiorgis, Computer Aided Chemical Engineering; published by Elsevier, 2018.

The continuous synthesis of (*S*)-warfarin was demonstrated by Porta et al. (2015), featuring the nucleophilic addition of 4-hydroxy-coumarin to benzalacetone in the presence of TFA and a chiral amine catalyst in 1,4-dioxane [67]. Upon addition of the candidate LLE solvent, the process forms an organic (product) phase containing recovered API and an aqueous (waste) phase. Several candidate separation solvents are compared for continuous LLE: ethyl acetate (EtOAc), isopropyl acetate (iPrOAc) and isobutyl acetate (iBuOAc). The CPM flowsheet for warfarin is shown in Figure 14 [68].

#### 3.1.5. Atropine

**Figure 15:**
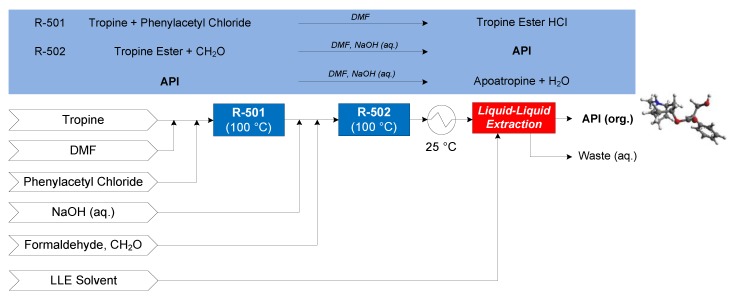
CPM flowsheet for atropine: flow synthesis [69] + continuous LLE [70]. Reproduced with permission from Diab and Gerogiorgis, AIChE Journal; published by John Wiley and Sons, 2019.

The continuous flow synthesis of atropine was demonstrated by Bédard et al. (2016), featuring two flow reactions: the esterification of tropine (in dimethylformamide, DMF) and neat phenylacetyl chloride at 100 °C (in R-501) to form tropine ester HCl, the free form of which is formed by the addition of sodium hydroxide (NaOH (aq.)). In R-502, the aldol addition of formaldehyde (CH_2_O) to the tropine ester at 100 °C under basic conditions forms the API, accompanied by an undesired elimination of API to apoatropine via condensation [69]. A subsequent continuous LLE in a cascade of vessels is performed with either diethyl ether (Et_2_O), *n*-butyl acetate (BuOAc) or toluene (PhMe) for comparative evaluation purposes [70]. The CPM flowsheet for atropine is shown in Figure 15.

#### 3.1.6. Nevirapine

**Figure 16:**
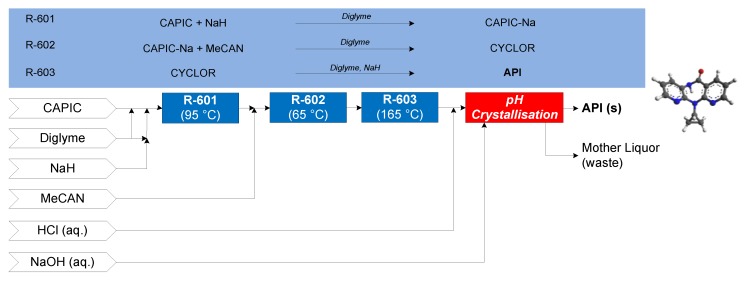
CPM flowsheet for nevirapine: flow synthesis [71] + continuous crystallization [72]. Reproduced with permission from Diab et al., Organic Process Research & Development; published by American Chemical Society, 2019.

The continuous flow synthesis of nevirapine was demonstrated by Verghese et al. (2017). First, 2-chloro-3-amino-4-picoline (CAPIC) and sodium hydride (NaH) form CAPIC-Na salt in diglyme at 95 °C in R-601; the effluent enters R-602 with neat 2-(cyclopropylamino)nicotinate (MeCAN) at 65 °C to form *N*-(2-chloro-4-methylpyridin-3-yl)-2-(cyclopropylamino)nicotinamide (CYCLOR). In the final reactor (R-603), CYCLOR flows over a packed bed of NaH to form nevirapine [71]. A subsequent purification and crystallization via pH change is performed to obtain purified API crystals. Different assumptions of solvent recovery, SR = {0%, 40%, 80%} (reflecting worst case, intermediate and laboratory-scale demonstrated recovery demonstrations, respectively), are considered. The CPM flowsheet for nevirapine is shown in Figure 16 [72].

The extent of modeling, simulation and optimization for the different API case studies considered vary: ibuprofen, artemisinin and diphenhydramine implement process simulation for design space investigation; warfarin and nevirapine studies implement Nonlinear Programming (NLP) for plantwide optimization for total cost minimization; atropine CPM implements Mixed Integer Nonlinear Programming (MINLP) for process synthesis to optimality, i.e., plant total cost minimization. Details of steady-state modeling, simulation and optimization implemented for each case study can be found in our previous research contributions listed above. Table 1 summarizes design option details for the different processes.

### 3.2. Plant Design Performance Metrics

Process performance metrics encompassing technical performance, process intensity and costs are compared for different APIs and selected separation option. The process metrics considered for the comparative evaluation presented here are: Plantwide API recovery; Mass Productivity, MP = Mass of Product / Total Mass in Process (a measure of how efficiently material is used in a process [73]); Number of reaction and separation stages—a measure of process intensity; Capital (CapEx) and Operating (OpEx) Expenditures per unit mass of API produced.

**Figure 17:**
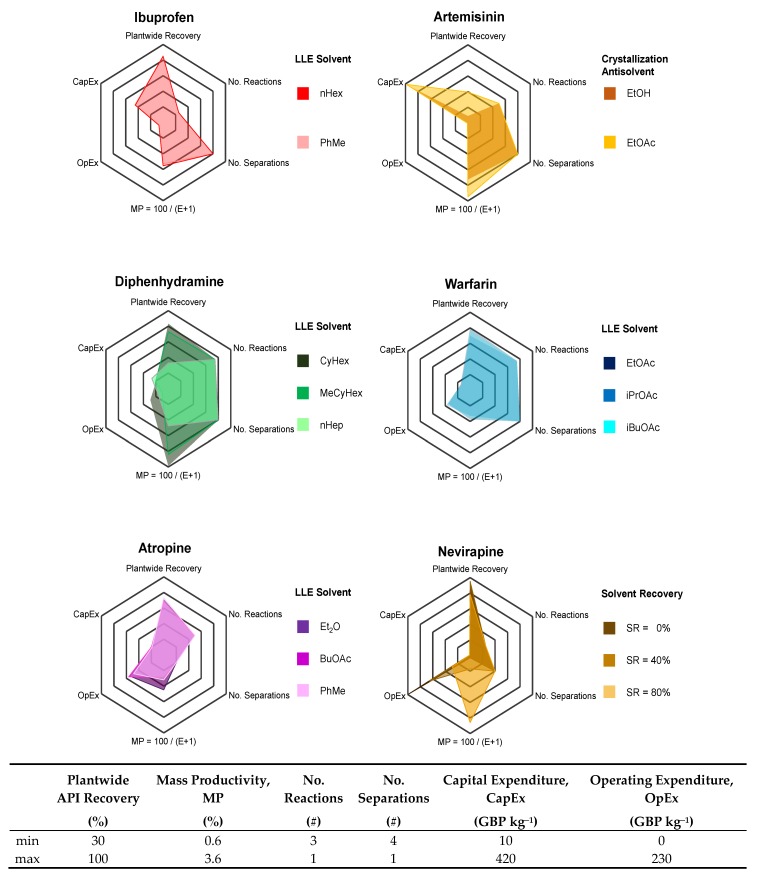
Performance metrics of various CPM processes for different APIs.

The process metrics for each API case and design option are listed in Table 2 and illustrated for comparative evaluation via a radar plot in Figure 17. Each axis (process performance metric) in Figure 17 bears a different meaning depending on whether it has a high or low value. Clearly, high plantwide recoveries and MP but lower cost components are desirable. For the number of reaction and separation stages, reverse-ordered axes are used to illustrate that lower values are preferable (i.e., fewer unit operations equate to lower process complexity). The greater total surface area that a design option covers in Figure 17, the better the process design is; it is also important that a design is sufficiently high in all categories, not just highly performing in a few. For each API, the number of reactions and separation stages have the same coordinates for each different separation option.

For ibuprofen, the different separation options (LLE solvent = {PhMe, nHex}) give similar results and thus the LLE solvent with the lower environmental/EHS impact (PhMe) is preferred [74]. For warfarin, each LLE solvent performs comparably, but has similar EHS characteristics; solvent selection should thus be informed by subsequent crystallization process design and the possibility for solvent harmonization, recovery and recycling.

For artemisinin, plantwide performance varies more significantly with antisolvent choice. The greater difference can be attributed to the different thermodynamic behaviors of the two antisolvents with the inlet mixture (toluene) due to the different polarities and functional groups on each antisolvent. For artemisinin, EtOH as antisolvent allows for lower costs and is more environmentally friendly than EtOAc; thus EtOH is the better antisolvent choice.

Similarly for diphenhydramine, the different separation performances between the different LLE solvent choices is due to the different thermodynamic behavior of the ternary system and hence phase splitting and API partitioning between the resulting organic (product) and aqueous (waste) phases; this is also due to the differences between the molecular structure of the LLE solvent choices. For diphenhydramine, nHep has both poorer EHS characteristics than either CyHex or MeCyHex as well as incurring higher costs; thus, either of the cycloalkane solvent choices is preferable.

For warfarin, the performances between different LLE solvent choices is comparable due to the similar thermodynamic behaviors of the ternary systems. For atropine, the LLE solvent choices perform comparably despite their different molecular structures, but Et_2_O and PhMe are less favorable than BuOAc with respect to their EHS characteristics; as for warfarin, consultation with processing requirements downstream and for plantwide operation + material efficiency is required. For nevirapine CPM, various values of Solvent Recovery (SR) are considered; whilst high SR (=80%) is attainable in laboratory-scale conditions, lower values are likely to be possible at larger scale operation. The assumed SR drastically affects OpEx, which is a significant contribution towards total costs, i.e., OpEx >> CapEx.

Pharmaceutical manufacturing is typically quite intensive in terms of material and energy consumption due to the multistep synthetic routes required to synthesize APIs as well as strict quality requirements which must be met prior to human consumption. Molecular Complexity Indices (CIs) are often used to quantify the complexity/difficulty to synthesize a molecule with respect to its structure. The most popular metric is the Bertz CI, which varies with the different numbers and types of functional groups and their interconnections [75,76]. 

**Figure 18:**
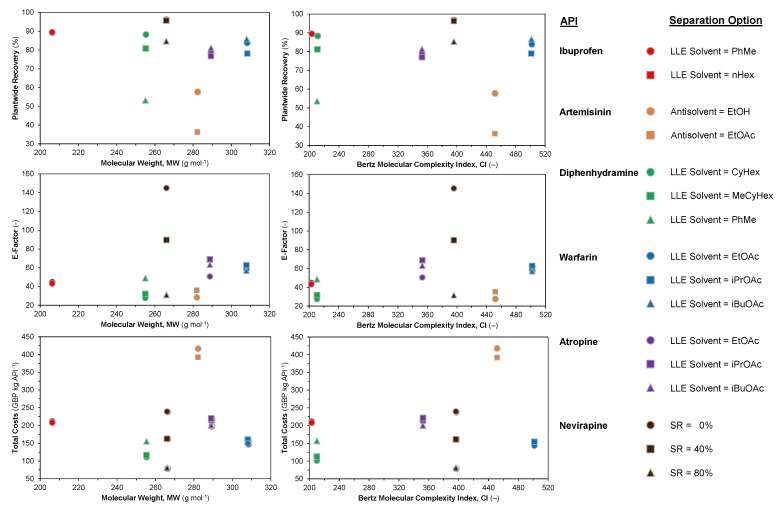
Performance metrics of various CPM processes for different APIs vs. Molecular Weight (MW) and Bertz Complexity Index (CI).

Our previous work has established correlations between complexity and economic parameters for a large set of top selling antibiotics [77,78]. Here, we examine the different performance metrics considered in Section 3.2 vs. their respective Molecular Weights (MWs) and CIs. Figure 18 shows plantwide recovery, E-factor and total costs vs. MW and CI for different API cases. For the dataset considered here, there is no obvious correlation between the performance metrics and MW/CI. Despite this, there are some observations to be made. The lowest plantwide recovery (by API) is artemisinin, which also has the highest costs. Designs for the considered APIs in this study have typical plantwide recoveries = 70–90% (with some outliers) and varying E-factors (*E* = 20–80), which are all either good or reasonable for pharmaceutical manufacturing [79,80,81]. This highlights that beyond this API recovery that cost benefits are incremental at best. Nevirapine has significantly higher E-factors than other APIs due to the purification implemented prior to crystallization via pH change, as described in the original literature studies [71,72].

These results illustrate that some of these CPM processes are leaner/further developed than others, i.e., there are still process improvements to be made with respect to cost reductions and plant efficiencies. It should be noted that different methodologies have been applied for different API cases (see Table 1) when comparing the design solutions presented here for different APIs and separation options; nevertheless, the results presented in this study illustrate different attainable regions of plantwide performance typical of CPM for the considered APIs, which have been highlighted as amenable to CPM success in both their flow synthesis and modeling demonstrations.

**Figure 19:**
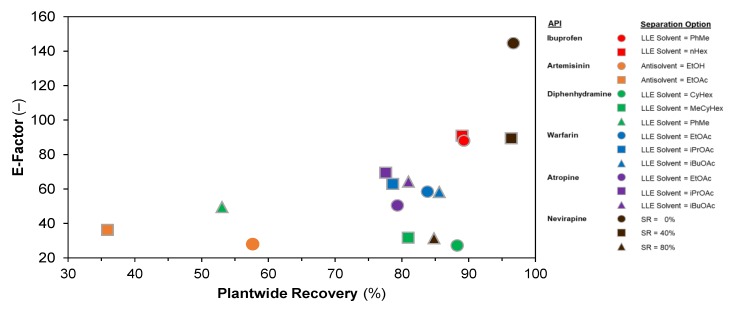
Plantwide E-factors vs. attained API recoveries for different design cases.

Figure 19 compares the attained E-factors (a measure of material efficiency) vs. plantwide recoveries. For ibuprofen, the attained recoveries, and thus E-factors, are similar for both LLE solvent choices (nHex, PhMe). For artemisinin, diphenhydramine and warfarin, the E-factor decreases (i.e., material efficiency improves) as plantwide recovery increases—this is expected, as waste quantities are lower when the plant API recovery is high for a specified plant API capacity. For atropine, the same trend is not observed; this is due to different quantities of separation solvent being used between design cases in order to attain total cost minima in the design cases [70]. For nevirapine, the different design cases correspond to different solvent recovery assumptions; evidently, as SR increases, the E-factor improves (i.e., decreases).

### 3.3. API Cost Component Contributions

**Figure 20:**
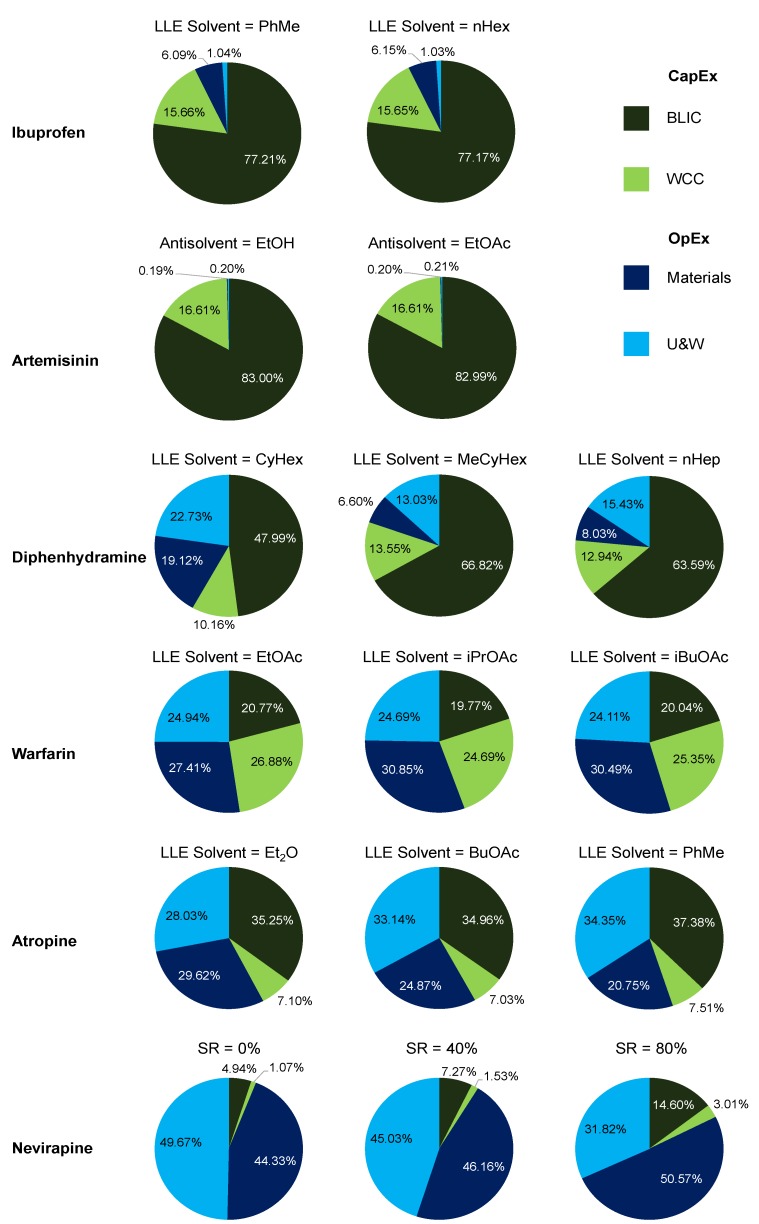
Total cost contributions towards API production.

Figure 17 shows overall API cost contributions comparatively. Figure 20 shows the cost component contributions on a more detailed level to gain deeper insight into API cost contributions and how these are related to the design options selected from our previous studies. CapEx contributions are the Battery Limits Installed Cost (BLIC) and Working Capital and Contingency (WCC); OpEx contributions are materials and Utilities + Waste (U&W) [63].

For ibuprofen, total cost components are dominated by CapEx, which is in turn predominantly BLIC components for both LLE solvents. Similar results are also observed for artemisinin, which implements antisolvent crystallization. For artemisinin, OpEx contributions are so low due to the main feedstock, DHAA, being a waste product from an existing process and considered to have negligible costs in its acquirement in comparison to the other material prices [63,64].

For diphenhydramine, OpEx contributions are more significant than for ibuprofen and artemisinin. Greater LLE solvent usage was used for the diphenhydramine design cases (in terms of the mass ratio of separation solvent-to-incoming feed stream) than for ibuprofen and artemisinin. The OpEx contributions for MeCyHex are lower than for CyHex due to its lower material price and similar recovery (and thus flowrates and equipment sizes) [66]. The CapEx contributions for this API are less impactful due to less equipment being used, i.e., only one synthesis and one separation stage for diphenhydramine [65]. Process intensification and simplification is an excellent way to reduce costs and streamline production. Similar trends are observed for both warfarin and atropine, with components being similar across different separation options due to their similar performances (i.e., recoveries).

For nevirapine, total OpEx components reduce with increasing Solvent Recovery (SR) assumption due to less fresh solvents being required. The values of SR considered are 0% (worst case scenario = no recovery), 40% (intermediate) and 80% (best case scenario = recovery attained in the laboratory-scale demonstration [71]); other values can easily be compared to these results using the published plantwide model and optimization framework [72].

Total cost components (i.e., CapEx and OpEx) have been scaled per unit mass of API produced in the product streams of each upstream CPM plant for fair comparison where different plant capacities are considered in different studies. Each case study considered upstream plant total costs as the economic metric for comparative evaluation of different process designs. Comparison of optimal Net Present Values (NPVs) can also provide valuable insight and alternative process designs for different APIs, but are subject to API sales price variation, which may be quite significant for certain drugs (e.g., artemisinin). Ultimately, when choosing whether to switch to continuous operation, clear operational and economic benefits must be clear over traditional/current manufacturing methods for the API.

## 4. Conclusions

Design space investigation of CPM is a useful task in elucidating the attainable regions of operation and process efficiency and attainable product quality. The literature contains many demonstrations that have elucidated operating regions and mapped design spaces on a technical basis at unit operation level for API synthesis, purification and downstream formulation, but not integrated stages thereof. In this study, we compare technoeconomic plantwide analyses for upstream CPM (reaction + separation) for various APIs considered by our group, all of which have high economic impact and societal importance. The design space investigation for each API considers reaction + purification/separation, with the main tuning parameters between design cases pertaining to the separation processes, which have receive little attention in comparison to the number of literature studies on synthesis optimization. Comparative evaluation of different design cases is on the basis of technical, operational, economic and EHS criteria. Currently, decisions on whether to operate continuously is made on a case-by-case/API basis. Elucidating operating regions for demonstrated CPM for different APIs is an important step towards more systematic selection and screening of promising candidates for continuous production.

## Figures and Tables

**Table 1:** Summary of separation design options for each API case study.

API	Application	Separation	Option	No. Stages		Refs.		Methodology
				Synth.	Sep.	Synth.	Sep.	
Ibuprofen	Analgesic	LLE	PhMe	3	1	[62]	[63]	Simulation
			nHex	3	1	[62]	[63]	Simulation
Artemisinin	Antimalarial	Crystallization	EtOH	2	1	[64]	[63]	Simulation
			EtOAc	2	1	[64]	[63]	Simulation
Diphenhydramine	Antihistamine	LLE	CyHex	1	1	[65]	[66]	Simulation
			MeCyHex	1	1	[65]	[66]	Simulation
			nHep	1	1	[65]	[66]	Simulation
Warfarin	Anticoagulant	LLE	EtOAc	1	1	[67]	[68]	NLP
			iPrOAc	1	1	[67]	[68]	NLP
			iBuOAc	1	1	[67]	[68]	NLP
Atropine	Nerve agents	LLE	EtOAc	2	4	[69]	[70]	MINLP
			BuOAc	2	4	[69]	[70]	MINLP
			PhMe	2	4	[69]	[70]	MINLP
Nevirapine	HIV treatment	Crystallization	SR = 00%	3	3	[71]	[72]	NLP
			SR = 40%	3	3	[71]	[72]	NLP
			SR = 80%	3	3	[71]	[72]	NLP

**Table 2:** Summary of performance metrics for each API case study (listed in Table 1).

API	Separation	Option	Recovery (%)	E-Factor (−)	Cost Component (GBP kg^−1^)			Ref.
					CapEx	OpEx	Total	
Ibuprofen	LLE	PhMe	89.2	44.7	195.9	15.3	211.2	[63]
		nHex	89.5	43.2	192.7	15.2	207.9	[63]
Artemisinin	Crystallization	EtOH	36.1	35.1	389.6	1.5	391.2	[63]
		EtOAc	57.7	28.1	414.9	1.7	416.6	[63]
Diphenhydramine	LLE	CyHex	88.3	27.1	89.6	64.5	154.1	[66]
		MeCyHex	81.1	31.1	92.8	22.7	115.5	[66]
		nHep	53.1	48.2	118.4	36.3	154.7	[66]
Warfarin	LLE	EtOAc	85.7	57.1	71.5	78.5	150.0	[68]
		iPrOAc	78.8	62.2	68.4	85.4	153.8	[68]
		iBuOAc	84.8	58.4	66.7	80.2	146.9	[68]
Atropine	LLE	Et_2_O	79.3	50.4	90.1	122.6	212.7	[70]
		BuOAc	77.8	68.0	91.8	126.7	218.5	[70]
		PhMe	81.0	62.9	88.5	108.7	197.2	[70]
Nevirapine	Crystallization	SR = 0%	86.7	144.5	14.3	223.6	237.8	[72]
		SR = 40%	86.4	89.0	14.2	147.3	161.5	[72]
		SR = 80%	85.0	30.5	13.8	64.5	78.3	[72]
